# The INSPIRE Registry: Entering a New Era of Medical Device Research in the Neurovascular Field

**DOI:** 10.1007/s00062-020-00981-4

**Published:** 2020-12-10

**Authors:** István Szikora, Markus Holtmannspötter, Mario Martinez-Galdamez, Saleh M. Lamin, Laurent Spelle, Francis Turjman, Jens Fiehler, Steering Committee

**Affiliations:** 1 National Institute of Clinical Neurosciences, Semmelweis University Budapest, Budapest, Hungary; 2 Dept. of Neuroradiology, Klinikum Nürnberg, Paracelsus Medical University, Nürnberg, Germany; 3 Neuroradiology Unit, University Clinical Hospital of Valladolid, Valladolid, Spain; 4 Neuroradiology, Queen Elizabeth Hospital Birmingham, Birmingham, UK; 5 NEURI Brain Vascular Center, Bicêtre Medical Center, Paris-Sud University, Paris, France; 6 Department of Interventional Neuroradiology, Lyon University Hospital, Lyon, France; 7 Department of Diagnostic and Interventional Neuroradiology, University Medical Center Hamburg-Eppendorf, Hamburg, Germany

Following the great revolution of medicine by efficient pharmaceutical products in the late 1900s, we are now experiencing a new revolution by a flood of new medical devices in the early twenty-first century. Although this new revolution affects almost every specialty in medicine, it has a particularly significant role in the neurovascular field. The introduction of thrombectomy into the treatment of acute ischemic stroke (a therapy strictly dependent on medical devices) is so far considered the twenty-first century’s most significant breakthrough in medicine. Altogether, the impact of devices and technology as well as the industry behind them, is growing by the hour. While new devices coming onto the market are greatly widening the capacity to effectively cure diseases that were untreatable just some years ago, the huge number of new techniques can make finding the best therapeutic choice difficult for physicians. Differentiating marketing slogans from scientific evidence is becoming a challenge for the medical community. Moreover, the industry is commonly accused of being run by only commercial interests. Frequently, the medical community has the first experience with a new technique after it has already been commercialized based on the safety profile but without demonstrating its true therapeutic value to patients.

Understanding the fast-growing impact of medical devices in real-world medicine and acknowledging the shared responsibility of device producers and the medical community, an innovative new study platform has been recently introduced: the Innovative Neurovascular Product Surveillance Registry (INSPIRE, ClinicalTrials.gov Identifier: NCT02988128). The INSPIRE is a unique, large scale, multitherapy, long-term observational study platform, sponsored by Medtronic Neurovascular (Irvine, CA, USA). This registry is designed to continuously monitor the safety and performance of any newly commercialized device that is introduced into the neurovascular field by the sponsor. As part of INSPIRE, patients are being prospectively enrolled from a large number of selected sites worldwide, whenever treated with any of the sponsor’s new neurovascular device(s). To ensure real world experience and to reduce bias, all study data are based on patients treated by the device based on routine standard of care. Site data are monitored and safety event data are assessed by an independent clinical events committee, while efficacy is assessed by an independent central core laboratory. Data on consecutively treated patients provide a large volume database with real-world evidence that is helping physicians to optimize therapeutic strategies by finding the best treatment paradigms based on scientific evidence. These data may further help the sponsor in understanding the patterns of product use, in allowing decision makers to establish reimbursement rules, and facilitating the decision-making processes for hospital managers and healthcare payers to choose the best options for their practice. Finally, such studies are in line with recent trends in medical device regulations, such as the directive 2017/745 of the European Parliament and Council that sets out standards of postmarketing clinical surveillance for medical device manufacturers.

The true value of this new study platform is soon to be seen, as the first chapter of INSPIRE will be debuted with over 700 patients enrolled across 30 international sites following treatment with the latest models of flow diverter devices, the Pipeline™ Flex and Shield (Medtronic Neurovascular, Irvine, CA, USA) devices. The 1‑year follow-up results of the first 100 patients treated with Pipeline™ Flex with Shield technology™ have been presented at the ESMINT congress in September 2020 and this arm of the study has received official endorsement from the European Society of Minimally Invasive Neurological Therapy (ESMINT) in 2020.

Regarding hemorrhagic stroke, a subset of 12 French sites have completed the enrollment of 184 patients treated with the Pipeline™ device to support the renewal of the reimbursement dossier. The second chapter of INSPIRE is focusing on the sponsor’s new devices for ischemic stroke. Patients treated with Solitaire^TM^ X and React^TM^ 68/71, will be enrolled in the INSPIRE STROKE registry.

This new approach to postmarketing real-world data collection is based on a strong cooperation between industry and the neurovascular medical community, and carries a great potential in improving patient safety, treatment efficacy and physician’s trustfulness in both product and manufacturer.

